# Spiers Memorial Lecture: Engineering biocatalysts

**DOI:** 10.1039/d4fd00139g

**Published:** 2024-07-24

**Authors:** Donald Hilvert

**Affiliations:** a Laboratory of Organic Chemistry, ETH Zürich 8093 Zürich Switzerland hilvert@ethz.ch

## Abstract

Enzymes are being engineered to catalyze chemical reactions for many practical applications in chemistry and biotechnology. The approaches used are surveyed in this short review, emphasizing methods for accessing reactivities not expressed by native protein scaffolds. The successful generation of completely *de novo* enzymes that rival the rates and selectivities of their natural counterparts highlights the potential role that designer enzymes may play in the coming years in research, industry, and medicine. Some challenges that need to be addressed to realize this ambitious dream are considered together with possible solutions.

## Abstract 2

Enzymes are being engineered to catalyze chemical reactions for many practical applications in chemistry and biotechnology.

## Graphical abstract

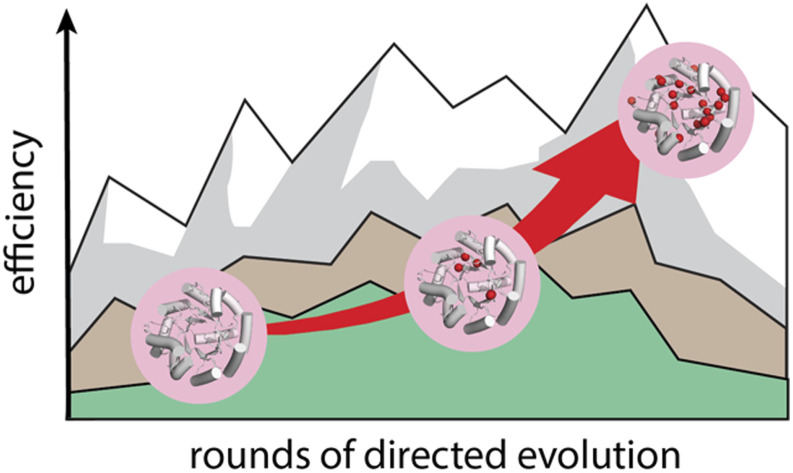

## Introduction

For millennia, mankind has harnessed the power of enzymes for baking, brewing and sundry other purposes. Although such applications remain relevant, our understanding of what enzymes are and how they function has advanced substantially. Today, these agents serve as highly active and selective catalysts in many areas of biology and chemistry.

The properties of enzymes that make life possible—impressive catalytic efficiency and precision—lend themselves to applications far beyond their normal biological roles. Ready access to enzymes capable of copying, cutting, and splicing DNA enabled the biotechnological revolution, for example. Other enzymes are employed directly as therapeutic agents to treat diseases like leukemia (asparaginase^1^), severe combined immunodeficiency (adenosine deaminase^2^) and phenylketonuria (phenylalanine hydroxylase^3^). Biocatalysts have also been recognized as pivotal for a greener, more sustainable chemical industry.^4,5^

Although natural enzymes are remarkable catalysts, they are often poorly suited for non-natural applications due to challenges in production, substrate specificity, or stability. Robust methods are consequently needed to tailor their properties or, more ambitiously, create biocatalysts with entirely new activities. In this short review, common methods for tackling these issues are highlighted.

## Enzyme engineering and evolution

The success of an enzyme application hinges on identifying a suitable catalyst for the reaction of interest. Genomic databases are one source of natural enzymes; strain collections and metagenomic libraries are another.^4,5^ For some applications, these proteins can be implemented directly. For others, engineering may be required. If structural and mechanistic information is available, rational approaches can successfully modify their properties. Often, a few mutations suffice to achieve the desired features.^6^ In other instances, extensive remodeling of the enzyme may be necessary.

**Fig. 1:**
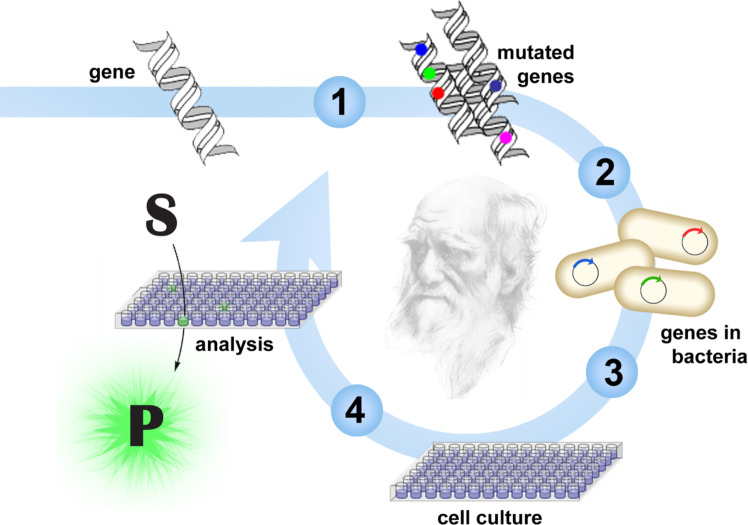
Typical workflow for directed evolution. The process involves gene diversification (1), transformation of bacteria cells (2), growing up individual variants in microtiter plates (3), and screening the variants, for example with a fluorogenic substrate (4). Iterative rounds of mutagenesis and screening allow gradual optimization of catalyst properties.

Approaches that mimic Darwinian evolution are well suited for demanding engineering challenges (Fig. 1).^7–11^ In a directed evolution experiment, the gene for the enzyme of interest is randomized to create DNA libraries. Following *in vitro* or *in vivo* production of the encoded variants, those with improved properties are identified by screening or selection, and their respective genes are amplified for characterization or further diversification. As in natural evolution, beneficial mutations accumulate over iterative mutagenesis and screening/selection cycles, resulting in gradual catalyst optimization.

Over the last three decades, the rates and substrate preferences of many biocatalysts have been engineered by directed evolution. This methodology is also useful for enhancing a protein’s physical characteristics, including expression yield, thermal stability, and tolerance to organic solvents, which may be important for large-scale manufacturing processes. All that is typically needed for a successful evolutionary campaign is for the starting protein to exhibit at least low starting activity for the desired transformation.

If non-native reactivity is targeted, the ability of an enzyme to catalyze reactions unrelated to its normal biological function, also called catalytic promiscuity,^12^ may provide a solution. The catalysis of carbene and nitrene transfer reactions by cytochrome P450 and other heme proteins is a case in point.^13^ These reactions proceed *via* metal–carbenoid or metal–nitrenoid intermediates mechanistically related to the native ferryl–oxo intermediates involved in oxygen insertion into unactivated C–H bonds. Diversification of this promiscuous activity by directed evolution has produced families of enzymes that catalyze cyclopropanations, intramolecular C–H aminations, intermolecular amination of benzylic C–H bonds, N–H insertions, aziridinations, and sulfimidations, usually with good total turnovers and excellent stereoselectivity. Biocatalytic routes to enantiopure organosilicon and organoboron compounds by heme-mediated carbon–silicon^14^ and carbon–boron^15^ bond formation are also notable in this context.

Common biological cofactors have been discovered to be another source of reactivity unexplored by natural evolution. For instance, the photoexcited states of NAD(P)H and FMN have been shown to facilitate electron transfer to substrates bound within enzyme active sites, enabling enantioselective radical dehalogenation reactions by NADPH-dependent ketoreductases^16^ and asymmetric hydro-dehalogenations of α-bromoesters by flavin-dependent ene-reductases.^17^ The mechanistic similarities between radical-relay reactions from synthetic chemistry and the native reactions of nonheme iron enzymes have similarly inspired efforts to enzymatically convert synthetic radical precursors into highly reactive species such as nitrogen-centered radicals that then undergo abiological reactions with high selectivity.^18,19^ In another recent example, a pyridoxal 5′-phosphate (PLP)-dependent enzyme has been combined with photoredox catalysis to create a novel radical route to diverse noncanonical amino acids, including those containing multiple chiral centers, without the need for protecting groups.^20^

## Biocatalysis for industry, medicine, and the circular economy

**Fig. 2:**
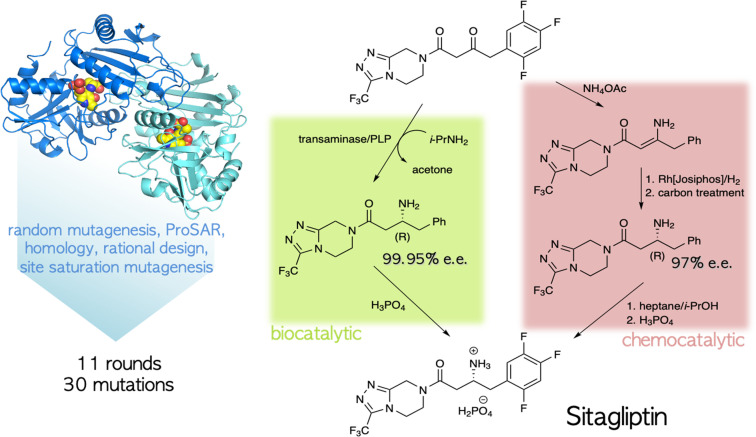
Biocatalytic *vs.* chemocatalytic routes to the anti-diabetic medication sitagliptin.^21^ The PLP-dependent transaminase was extensively mutagenized to meet the specifications of the industrial process.

**Fig. 3:**
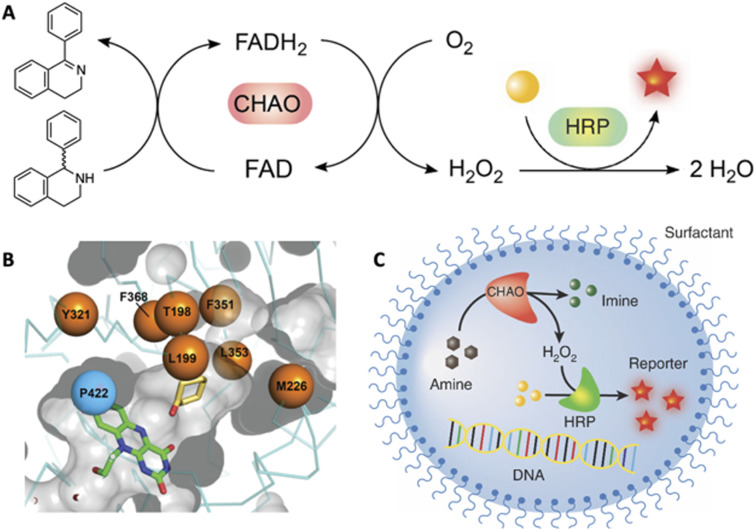
High-throughput evolution of cyclohexylamine oxidase (CHAO) for the stereoselective oxidation of 1-phenyl-1,2,3,4-tetrahydroisoquinoline, a precursor of the blockbuster drug solifenacin. (A) The coupled assay used to monitor the reaction. (B) The CHAO active site showing the eight residues that were mutagenized to create a 1.7 × 10^6^-membered library, which (C) was exhaustively screened by droplet-based microfluidics to identify a highly active and stereoselective variant.

The ability to tailor enzyme properties reliably has made biocatalytic production of small-molecule drugs an increasingly attractive alternative to traditional synthetic chemistry. The creation of stereoselective biocatalysts to generate chiral amines, critical components of many pharmaceuticals, is illustrative. The successful redesign of a pyridoxal-dependent ω-transaminase for the industrial-scale production of sitagliptin, a blockbuster antidiabetic drug, was a watershed event in this regard (Fig. 2).^21^ Not only was the enzymatic process superior to a previously established route involving a rhodium-based chiral catalyst, but the evolved enzyme displayed an unexpectedly broad substrate scope, rendering it an effective catalyst for the reductive amination of numerous prochiral ketones. Building on this success, many industrially relevant oxidoreductase enzymes for the synthesis of chiral amines, including monoamine oxidases (MAOs), amine dehydrogenases (AmDHs), imine reductases (IREDs) and reductive aminases (RedAms), have been engineered.^22,23^ If product formation can be linked to a fluorescent readout, either directly or through a coupled reaction, ultrahigh-throughput microfluidic screening can dramatically accelerate the optimization process (Fig. 3).^24^

Biocatalysis has much to offer the burgeoning field of synthetic biology as well. Efforts to expand the genetic code, for example, have relied on engineered aminoacyl-tRNA synthetases (aaRSs) that selectively aminoacylate suppressor tRNAs with more than 250 noncanonical amino acids.^25,26^ Adding such enzymes to the translational machinery of bacterial and eukaryotic hosts has enabled the production of recombinant proteins containing unique chemical functionality, opening exciting avenues in protein biochemistry and cell biology. In another area, CRISPR nucleases, such as Cas9, have been engineered to improve RNA-guided genome editing technologies.^27^ These efforts have afforded tools for highly specific base editing, regulation of gene expression, and genome imaging. Notably, phage-assisted continuous evolution enables the optimization of these enzymes on much faster timescales than conventional methods.

Global environmental concerns are also being addressed through biocatalysis. In this context, enzymatic degradation of plastic followed by polymerization of their constituent building blocks or valorization into other products has received considerable attention as a means of waste recycling. Natural hydrolytic enzymes that catalyze the breakdown of polyethylene terephthalate (PET) plastics have been evolved in the lab to improve both efficiency and thermostability for commercial applications. One variant, called HotPETase, rapidly depolymerizes semicrystalline PET and selectively decomposes the PET-containing components of laminated materials.^28^ Microwave pre-treatment has been found to accelerate such enzymatic depolymerization reactions substantially, likely by making the polymer chains more accessible to the enzyme.^29^ Recycling enzymatically depolymerized PET waste has also yielded PET polymers that exhibit the same properties as petrochemical PET,^30^ supporting the feasibility of a circular carbon economy for these plastics.

## Biocatalytic pathways, cascades, cells and systems

**Fig. 4:**
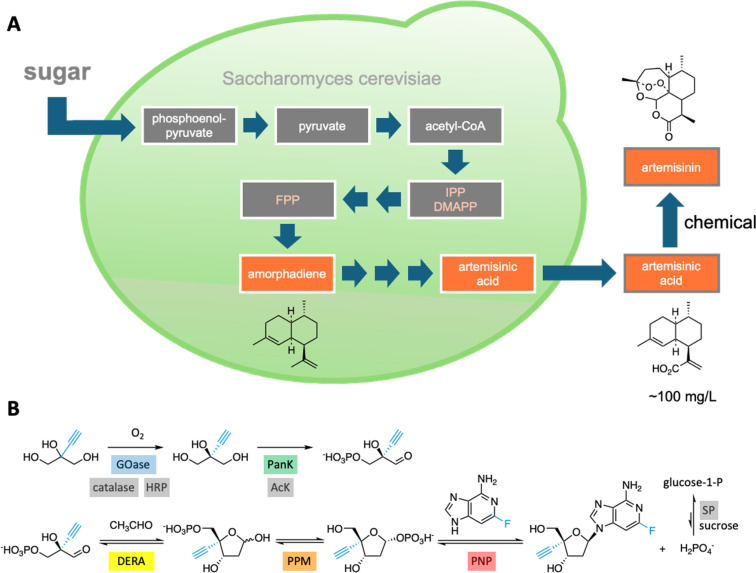
Natural and artificial enzymatic cascades. (A) An engineered pathway in yeast cells for the production of artemisinic acid, a precursor of the malaria drug artemisinin.^32^ (B) An *in vitro* enzymatic pathway to the HIV drug islatravir uses nine enzymes, five of which were engineered to accommodate the non-natural substrates (highlighted in color).^36^

Integrating sequentially acting enzymes into synthetic reaction cascades, either *in vivo* or *in vitro*, is an effective strategy for producing complex, high-value chemicals under mild conditions.^31^ Natural biosynthetic pathways have been successfully engineered to enhance the production of valuable natural products like the malaria drug artemisinin^32^ (Fig. 4A) and, more recently, a plant-derived glycosylated triterpenoid used as a potent vaccine adjuvant.^33^

Biocatalysts from different organisms can even be combined to create hybrid pathways. The construction of an artificial CO_2_ fixation cycle consisting of 17 enzymes from nine different organisms illustrates how complex such systems can be.^34,35^ This pathway includes an efficient enoyl-CoA carboxylase/reductase for the central carboxylation step to circumvent limitations associated with natural RuBisCo-based systems. Its efficiency after optimization, which included engineering the rate-limiting enzyme, exceeded that of the Calvin cycle. Embedding such pathways in living organisms could conceivably yield whole-cell biocatalysts capable of capturing atmospheric CO_2_ as a carbon feedstock.

In principle, engineered proteins can be used to construct completely artificial pathways. This was the case for a multistep enzymatic cascade developed for manufacturing the HIV drug islatravir (Fig. 4B).^36^ Five of the nine component enzymes were engineered by directed evolution to recognize non-natural substrates. The final pathway, which eliminated purification steps, recycled expensive cofactors, and coupled energetically favorable and unfavorable reactions, was more efficient and substantially shorter than alternative chemical routes.

Not all biocatalytic pathways involve discrete enzymes. The biosynthesis of many bioactive natural products is coordinated by gigantic multienzyme assemblies that function in an assembly-line fashion.^37^ Polyketide synthases^38^ (PKSs) and nonribosomal peptide synthetases^39^ (NRPSs) consist of repeating modules of several enzymatic domains, strung together like beads on a string, which are responsible for building block selection, activation, and subsequent oligomerization. Additional structural diversification can be achieved by specialized tailoring domains, and, once assembly is complete, the product is typically released by a terminal thioesterase. Because of their modularity, PKSs and NRPSs are appealing targets for engineering.

**Fig. 5:**
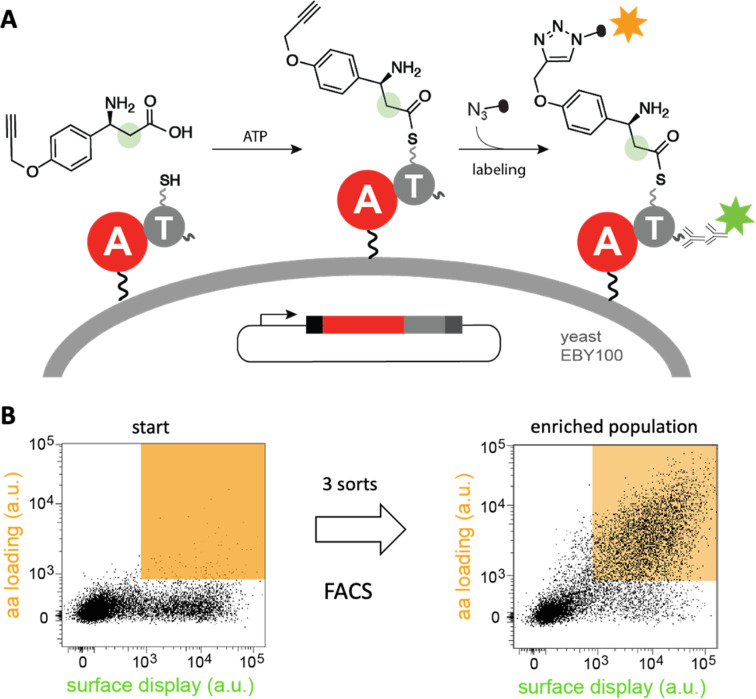
High-throughput engineering of an NRPS A domain. (A) A library of adenylation (A)-thiolation (T) didomains displayed on the surface of yeast were challenged with a non-canonical β-amino acid. If successfully activated by the A domain, the substrate is transferred to the pantetheinyl cofactor of the T domain and can be detected by “click”-labeling with a fluorophore. (B) Analysis of a library of tyrocidine A variants by fluorescence activated cell sorting (FACS) shows that variants that recognize β-amino acid (orange field) become enriched after three sorts.^41^

Diverse strategies for engineering these biosynthetic assembly lines have been investigated.^40^ In one approach, a recently developed yeast display system for screening millions of protein variants in a single experiment has been used to reprogram the substrate specificity of NRPS adenylation^41–43^ and condensation^44^ domains in high throughput (Fig. 5). The ability of the modified domains to process noncanonical building blocks such as β-amino acids, α-hydroxy acids, and fatty acids without compromising catalytic efficiency highlights its efficacy. Because such high-throughput strategies are likely general, they have the potential to facilitate the sustainable production of novel natural product analogs and other bioactive compounds.

## Artificial, biomimetic, and hybrid enzymes

Conferring completely new activities on a protein scaffold is generally more difficult than augmenting weak promiscuous activity. One of the easiest ways to access novel chemistry is to incorporate reactive functional groups into a binding pocket.^45^ For example, the protease subtilisin was converted into a peroxidase by chemically replacing its active-site serine with selenocysteine.^46^

Genetic code expansion has greatly extended this approach.^25,26^ Recombinant incorporation of noncanonical amino acids into proteins has been used to capture transient reaction intermediates, probe mechanisms, tune metal ion reactivity, and map long-range proton and electron transfer pathways.^47^ Catalysis of abiological reactions is also possible. Recent efforts in this direction include the creation of a photoenzyme that exploits triplet energy transfer to promote enantioselective [2 + 2] cycloadditions,^48^ a *p*-aminophenylalanine-containing enzyme that catalyzes condensation reactions of aldehydes with hydrazines and hydroxylamines *via* an iminium ion intermediate,^49^ and another that employs a genetically encoded arylboronic acid for the kinetic resolution of hydroxyketones by oxime formation.^50^ Notably, these reaction types have no equivalent in conventional homogeneous or enzyme catalysis. Moreover, because the responsible noncanonical amino acids are genetically encoded, the properties of the respective enzymes can be optimized by directed evolution.

Just as natural biochemical cofactors extend enzymatic capabilities, artificial prosthetic groups can also provide new-to-nature activities. This can be accomplished by replacing a natural cofactor like an iron heme with an abiological analog such as an iridium porphyrin. Myoglobin and P450 variants complexed with the iridium cofactor catalyze various carbene and nitrene insertion reactions,^51^ including carbene insertion into unactivated C–H bonds.^52^ Alternatively, cofactors can be attached either covalently or noncovalently to any protein to generate hybrid constructs, sometimes called semisynthetic enzymes,^45^ that combine the inherent reactivity of the cofactor with the protein’s molecular recognition properties. Binding biotinylated metal complexes to streptavidin, for example, is a rewarding route to artificial metalloenzymes for reactions like alkene hydrogenation, transfer hydrogenation of ketones, and olefin metathesis.^53^ Although rate enhancements are typically modest even after evolutionary optimization, these systems often exhibit high enantioselectivity. In another approach, amino acids like (2,2′-bipyridin-5yl)alanine can be incorporated genetically into an appropriate scaffold and have been used to position metal ions within binding pockets. Artificial copper-dependent enzymes prepared in this way have been shown to catalyze Diels–Alder cycloadditions, olefin hydration reactions, and Friedel–Crafts alkylations.^54^

## Computational enzyme design

State-of-the-art computational methods offer a powerful means of remodeling enzyme active sites for activities not found in nature.^55^ Conceptually analogous to catalytic antibody technology—which uses synthetic haptens to elicit immunoglobulins with binding pockets configured to catalyze a chemical reaction^56,57^—computational design begins with an idealized model of a minimal active site, consisting of a quantum mechanically calculated structure of the rate-limiting transition state(s) of the target reaction plus amino acid side chains spatially arranged to stabilize it.^58^ This ensemble is then docked into structurally characterized protein scaffolds *in silico*, linking the catalytic groups to the protein backbone, and repacking the active site to optimize interactions between the protein and the transition state. The resulting designs are ranked based on calculated energies, with the most promising candidates proceeding to experimental validation.

**Fig. 6:**
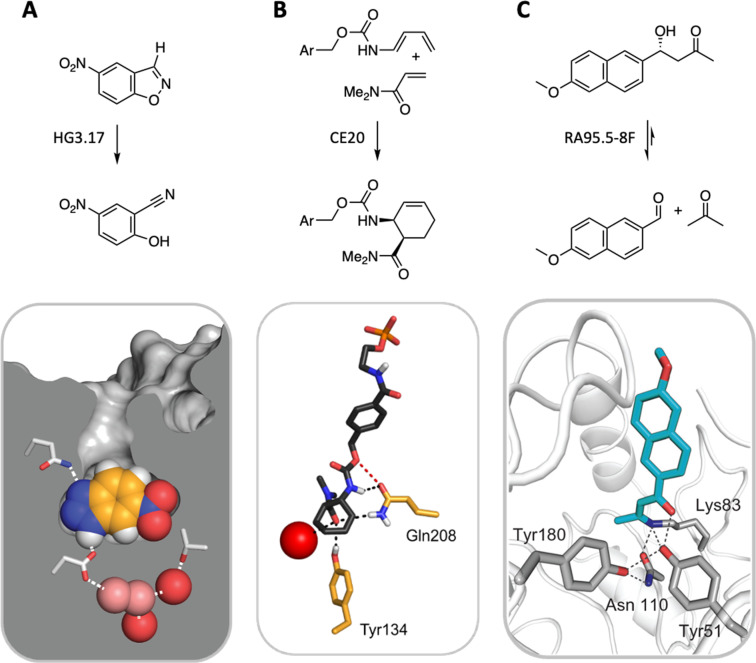
Three representative examples of computationally designed enzymes that have been evolved to high activity and selectivity by iterative rounds of mutagenesis and screening. (A) A Kemp eliminase for proton transfer from carbon,^62^ (B) a stereospecific Diels–Alderase,^65^ and (C) a multi-step aldolase that exploits amine catalysis.^68^

Computational enzyme design has yielded catalysts for a handful of mechanistically distinct chemical reactions (Fig. 6). These include ester hydrolysis,^59^ a unimolecular proton transfer reaction,^60–63^ a stereoselective Diels–Alder cycloaddition,^64,65^ and multistep aldol^66–68^ and Morita–Baylis–Hillman^69,70^ reactions. Although the initial computational designs exhibit modest efficiency, they could be readily optimized by directed evolution. The highest-performing catalysts exhibit activities that exceed those of comparable catalytic antibodies and, in the best cases, even rival the speed and stereoselectivity of natural enzymes, achieving rate accelerations exceeding a billionfold and producing products as single stereoisomers on a preparative scale. Based on structural and mechanistic analyses of the “winners,” the improvements achieved can be ascribed to substantial remodeling of the active site, installation of more complex arrays of functional groups, and/or minimizing unproductive states by changing protein conformation landscapes. Feeding these insights back into the design cycle may inform efforts to improve the starting computational design and thus minimize the need for time-consuming experimental optimization. Machine learning methods also offer exciting new computational tools for improving the properties of these systems.^71–75^

## *De novo* enzymes

Natural proteins offer a diverse range of architectures for enzyme engineering. Nevertheless, their evolutionary histories and complex sequence–structure relationships may complicate efforts to implant completely new activities. Because they are evolutionarily naïve, simplified *de novo* scaffolds can potentially circumvent such issues.

Because the rules for designing helical bundles are relatively well understood, they were among the first *de novo* scaffolds to serve as scaffolds for both receptors and catalysts.^76^ The assembly of heme-binding four-helix bundles that mimic the function of natural oxygen-binding proteins like myoglobin illustrates this approach.^77,78^ Another four-helix bundle protein featuring a hydrophobic binding pocket adjacent to a di-iron center catalyzes phenol oxidation,^79^ while a three-helix bundle, organized around a structural Hg(ii) ion and a catalytic Zn(ii) ion, functions as a carbonic anhydrase mimic.^80^ A zinc-containing supramolecular protein assembly was found to exhibit promiscuous β-lactamase activity.^81^ Hydrolytic activity was also installed in a thermostable α-helical barrel composed of seven helices arranged around a lumenal channel by introducing a Cys-His-Glu catalytic triad on the inward face of each helix.^82^

As an alternative to rational design, combinatorial libraries of binary-patterned four-helix bundles have proven to be a rich source of new chemical activities.^83^ The genetic selection of an esterase that rescues an auxotrophic *Escherichia coli* strain lacking a key gene for iron uptake in an iron-limited medium is impressive in this regard.^84,85^ This catalyst provides an enzymatic function that sustains the growth of a living organism using a sequence, structure, and mechanism that are dramatically different from those selected by nature to solve this specific biochemical challenge.

Recent progress toward computational design of atomically accurate proteins has greatly expanded the palette of *de novo* scaffolds, untouched by natural evolution, that can be utilized for enzyme design.^86^ In one example, retro-aldolase activity was conferred on a *de novo* eight-stranded β-barrel protein by using computational methods to install a reactive amine in a hydrogen-bonded network of functional groups, thus jump-starting innovation.^87^ However, the scaffold proved difficult to evolve, likely because its small size and extreme thermal stability hindered its ability to adapt to the different intermediates along the multistep reaction pathway. A closed α helical solenoid repeat protein, designed to bind heme in a reconfigurable pocket, proved more amenable to optimization and yielded a proficient peroxidase that reacts *via* a stable high-valent ferryl intermediate.^88^ More recently, a *de novo* TIM barrel scaffold that tightly binds lanthanide ions in its central cavity^89^ was found to activate molecules under mild conditions by cerium photoredox catalysis, catalyzing the radical C–C bond cleavage of aromatic and aliphatic 1,2-diols in aqueous solution, including lignin surrogates, upon irradiation with visible light.^90^

**Fig. 7:**
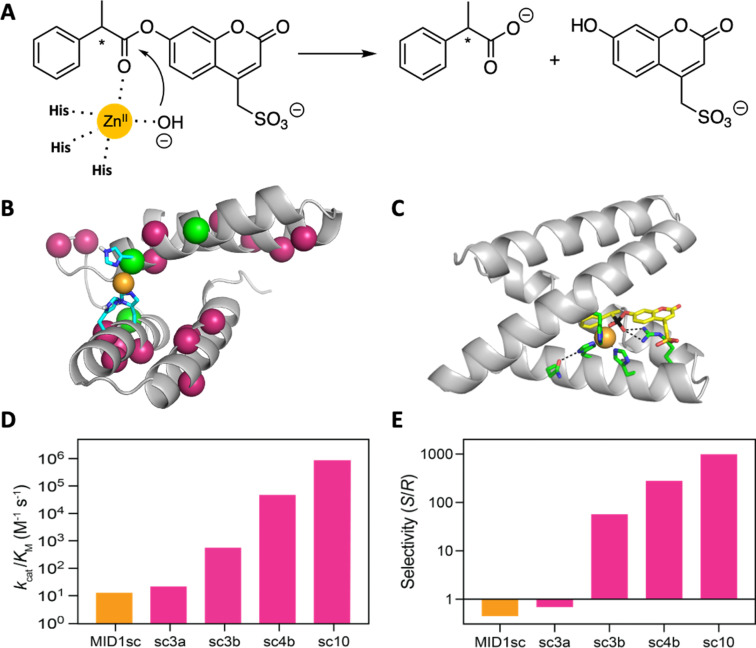
Design and evolution of a highly active and enantiospecific metalloesterase.^93^ (A) The computationally designed scaffold binds zinc and shows weak hydrolytic activity toward a coumarin ester of phenylpropionate. (B) Mutations (colored balls) introduced over 10 rounds of mutagenesis and screening are distributed throughout the protein scaffold. (C) A crystal structure of the optimized variant in complex with a transition state analog. (D) Activity increased by five orders of magnitude over the course of evolution, and (E) the stereospecificity first inverted and then increased in step with activity.

A computationally designed dimeric zinc-binding peptide called MID1 (metal interface design 1)^91,92^ has proven to be an unexpectedly versatile template for catalyst design (Fig. 7). Mimicking the biogenesis of primitive proteins, the two peptides were fused to generate a globular protein, and one of the two zinc sites was removed by replacing metal binding residues with non-coordinating amino acids suggested by computation.^93^ The single-chain version of the MID1 protein exhibited weak esterase activity, owing to an open coordination site on its zinc ion. This activity was improved >10 000 fold over ten rounds of directed evolution to afford an enzyme that cleaved a chiral ester substrate with remarkably high catalytic efficiency (*k*_cat_ = 1.6 s^−1^; *k*_cat_/*K*_M_ = 960 000 M^−1^ s^−1^) and stereospecificity (*E*_*S*/*R*_ = 1000). Indeed, the catalyst is orders of magnitude more efficient than other designed esterases and comparable to natural hydrolytic enzymes. A high-resolution structure of the optimized catalyst in complex with a transition state analog revealed the origins of these properties, highlighting an unexpected change in the coordination environment of the metal ion and the acquisition of an oxyanion-stabilizing arginine residue in the active site.^93^

Although conventional plate assays failed to yield further improvements in esterase activity, recent unpublished work has demonstrated that the enzyme’s efficiency can be increased by at least another order of magnitude (Oliver Allemann). This advance was achieved by exploiting an ultrahigh-throughput fluorescence-activated droplet sorting assay to screen libraries containing >10^6^ members. Because screening could be carried out at very high substrate concentrations ([S] ≫ *K*_M_), the improvement was largely manifest as a higher turnover number.

Strikingly, this small metalloprotein can also catalyze an abiological hetero-Diels–Alder reaction.^94^ In this case, modest starting activity was obtained through computational design and then improved by five orders of magnitude over multiple rounds of mutagenesis and screening. Detailed kinetic analysis showed that the optimized enzyme is highly stereoselective and >100 times more proficient than other known Diels–Alderases, including designed catalysts and natural enzymes involved in the biosynthesis of polyketide natural products. Its high activity can be ascribed to the use of a zinc cation, absent in the other catalysts, as a Lewis acid to activate the dienophile for reaction.

Simultaneous optimization of structure and function in a naïve scaffold not only illustrates how the first enzymes might have emerged from simpler precursors, but the high activities achieved also attest to the value of harnessing Lewis acids for new catalytic activities. Indeed, this helical bundle scaffold is remarkably malleable. It promiscuously catalyzes the reduction of azachalcones by Hantzsch esters, bimolecular Michael additions, and zinc-dependent retro-aldol reactions (Yusuke Ota, unpublished). Although starting activities are generally low, they can be increased substantially by multiple rounds of mutagenesis and screening. Changing the metal ion could conceivably provide a rich source of additional activities.

## Perspectives

Laboratory evolution and computational methods have become invaluable tools for engineering biocatalysts with customized activities and specificities. Not only can they adapt existing enzymes for new tasks, but they have also been key to realizing Emil Fischer’s 120-year-old dream^95^ of generating *de novo* enzymes that are as efficient and selective as their counterparts in nature. Being able to accelerate abiological reactions in this way has the potential to greatly expand the toolkit of biocatalysts available for chemical synthesis and other applications.

Nonetheless, many challenges persist. Producing new enzymes for energetically demanding transformations is still difficult, reflecting the complexity of the interconnected hydrogen-bonded networks needed to preorganize active site residues in catalytically competent orientations and to facilitate concerted interactions between side chains during catalysis. Even when design works, success rates and initial activities are low, pointing to inadequacies in the design tools that will require rectification.

The advent of deep learning technologies for predicting protein structure from sequence^96^ and “hallucinating” atomically accurate protein scaffolds^97–99^ will undoubtedly help address these issues. By learning how sequence maps to fitness, such methods will navigate vastly larger regions of sequence space than will ever be possible experimentally. The recent design of a *de novo* luciferase highlights the enormous promise of these approaches.^100^ To capture the dynamic properties of proteins, which is currently ignored in most design protocols, classical molecular dynamics (MD) and quantum mechanics/molecular mechanics (QM/MM) simulations may augment these efforts.^101,102^

Although time-consuming and costly, laboratory evolution can be expected to play a key role in enzyme design for the foreseeable future. As computational methods improve, though, its function should shift from fixing flawed designs to fine-tuning their properties. Advances in gene synthesis and next-generation sequencing have the potential to expedite the implementation and analysis of “smart” libraries,^103,104^ while (ultra)high-throughput screening methods^105^ can facilitate the search of large populations of variants and thus increase the likelihood of discovering synergistic combinations of rare but beneficial mutations. Recent initiatives to develop “self-driving laboratories” that explore protein fitness landscapes by integrating machine learning, decision-making, protein design, and experimental validation provide additional opportunities to automate and accelerate biocatalyst optimization.^106,107^

Protein design and evolution have made amazing progress in the last decade, signaling an exciting future for enzyme engineering in the years to come. Being able to design enzymes at will for any reaction you fancy will benefit efforts to address countless real-world challenges, paving the way to a more sustainable and prosperous future.

## Data availability

No primary research results, software or code were included, and no new data were generated or analyzed for this review.

## Author contributions

DH wrote this review article.

## Conflicts of interest

There are no conflicts to declare.
